# Use of easy measurable phenotypic traits as a complementary approach to evaluate the population structure and diversity in a high heterozygous panel of tetraploid clones and cultivars

**DOI:** 10.1186/s12863-017-0556-9

**Published:** 2018-01-16

**Authors:** Martin E. Tagliotti, Sofia I. Deperi, Maria C. Bedogni, Ruofang Zhang, Norma C. Manrique Carpintero, Joseph Coombs, David Douches, Marcelo A. Huarte

**Affiliations:** 1 Potato Research Group, Estación Experimental Agropecuaria Balcarce, Instituto Nacional de Tecnología Agropecuaria (INTA), Ruta 226 km 73.5, Balcarce, Buenos Aires, Argentina; 2 Consejo Nacional de Investigaciones Científicas y Técnicas–CONICET, Saavedra 15, C1083ACA Ciudad Autónoma de Buenos Aires, Argentina; 3 Potato Engineering and Technology Research Centre, Inner Mongolia University, 235 West College Road, Hohhot, China; 4 Department of Plant, Molecular Plant Sciences Bldg., Michigan State University, 612 Wilson Road #S148, East Lansing, Michigan MI 48824 USA

**Keywords:** Potato breeding, Phenotypic trait, Genetic diversity, SNP, Population structure

## Abstract

**Background:**

Diversity in crops is fundamental for plant breeding efforts. An accurate assessment of genetic diversity, using molecular markers, such as single nucleotide polymorphism (SNP), must be able to reveal the structure of the population under study. A characterization of population structure using easy measurable phenotypic traits could be a preliminary and low-cost approach to elucidate the genetic structure of a population. A potato population of 183 genotypes was evaluated using 4859 high-quality SNPs and 19 phenotypic traits commonly recorded in potato breeding programs. A Bayesian approach, Minimum Spanning Tree (MST) and diversity estimator, as well as multivariate analysis based on phenotypic traits, were adopted to assess the population structure.

**Results:**

Analysis based on molecular markers showed groups linked to the phylogenetic relationship among the germplasm as well as the link with the breeding program that provided the material. Diversity estimators consistently structured the population according to a priori group estimation. The phenotypic traits only discriminated main groups with contrasting characteristics, as different subspecies, ploidy level or membership in a breeding program, but were not able to discriminate within groups. A joint molecular and phenotypic characterization analysis discriminated groups based on phenotypic classification, taxonomic category, provenance source of genotypes and genetic background.

**Conclusions:**

This paper shows the significant level of diversity existing in a parental population of potato as well as the putative phylogenetic relationships among the genotypes. The use of easily measurable phenotypic traits among highly contrasting genotypes could be a reasonable approach to estimate population structure in the initial phases of a potato breeding program.

**Electronic supplementary material:**

The online version of this article (10.1186/s12863-017-0556-9) contains supplementary material, which is available to authorized users.

## Background

Selection pressure exerted to attain genetic improvement threatens the allelic diversity of crop genomes. Offspring are mainly derived from the elite varieties which generates a narrow genetic base [1, 2]. Genetic diversity analysis reveals the genetic backgrounds and relationships of crop germplasm and provides strategies to establish, utilise, and manage crop core collections [3]. In the same line, understanding taxonomy, phylogenetic relationships and genetic variability in crop varieties is critical for determining genetic relationships, characterising germplasm, controlling genetic erosion, designing sampling strategies or core collections, establishing breeding programs, and the registration of new cultivars. Crop diversity can be assessed through phenotypic and biochemical traits, pedigree analysis or by molecular markers [4–6] and is expected to find some degree of correlation among them taking into account that the molecular approach is the most unbiased. Among these approaches, phenotypic traits, as tuber size or skin colour, are intuitive and practical to assess in the selection process, although they are subject to environmental effects and selection pressure during domestication and breeding. The characterization of the correct population structure within germplasm collections is critical to identify and correctly interpret the associations between functional and molecular diversities [7, 8]. The traditional estimators of population structure will reduce the genetic information of germplasm if pre-defined populations do not accurately match the biological reality [9]. This is a serious problem for correct evaluation of the genetic structure and diversity, especially in a crop cultivated across a large geographical area [10]. Software like STRUCTURE [11] was created to detect genetic relatedness in a collection of genotypes with multilocus molecular markers information (e.g. SNP). Population structure has previously been examined in potatoes within an association mapping context by Simko et al. [12], D’hoop et al. [13] and Hirsch et al. [14].

Despite having many species and subspecies and being distributed in dissimilar environments, potato’s complex genetic nature has hindered the progress of traditional breeding efforts [10, 15]. Different approaches in potato breeding, as linkage and association mapping using genome wide selection or candidate genes, have been described rendering several quantitative trait loci (QTL) important for the regulation of agronomic traits [10, 16–19]. However, association studies need the assessment of phenotypic and genotypic variability [16]. Recently, phenotypic traits with high broad-sense heritability and its QTLs have been described in a potato panel [20].

Usually, high numbers of potato entries with insufficient data are periodically incorporated in breeding centres, either from germplasm collection trips or other from germplasm sources with specific traits of interest. At the beginning of the breeding process, the breeder has several entries to be included in a potato crossing panel with the expectation of having high genetic diversity. This purpose may be hampered by mislabelling, duplications, genetic closeness and environmental covariation, among other factors. Therefore, a preliminary approach with easy measurable phenotypic traits could be a reasonable tool to select genotypes with contrasting traits of interest at the moment of the onset of the potato panel. This is particularly important in developing countries where resources and molecular analysis instruments are not easily accessible for full genetic screening.

The aim of this work was to characterise and describe the structure of a population with a low time-cost strategy that uses easy measurable phenotypic traits as a preliminary screening before SNP genotyping. Simultaneously, we explored the potential of estimating genetic diversity using a joint phenotypic and molecular approach.

## Methods

### Plant material

The population assessed was composed by 183 potato accessions from diverse sources such as North and South America, Europe and Asia, varieties and clones from the International Potato Center (CIP) and the Argentine National Agricultural Technology Institute (INTA) -Balcarce breeding programs. Cultivated potato (*Solanum tuberosum* Gp. Tuberosum, Gp. Andígena and *S. stenotomun*), wild species (*S. chacoense*, *S. tarijense*) and interspecific hybrids genotypes (*S. tuberosum* Gp. Tuberosum var. Calen INTA x *S. gourlayii*) were represented in the studied germplasm. According to the taxonomy, ploidy level, breeding program and geographic provenance, a particular genotype was assigned to a priori groups (A-H). The list with the germplasm used in this study, the a priori group label, germplasm source, ploidy level, species and type of traits analysed are presented in Additional file 1.

The field trials were planted at the experimental station of INTA at Balcarce, Argentine (37° 45′32″S, 58° 17′´O), during two growing seasons (2011–2012, 2012–2013), under local standard phytosanitary, fertilization and irrigation regimes. The experimental design used was an augmented block design with eight blocks and eight standard varieties (Asterix, Bintje, Frital INTA, Innovator, Kennebec, Newen INTA, Pampeana INTA and Spunta) for both seasons [21, 22]. All plant material was planted in the field with healthy virus-free seed tubers. Five seed tuber pieces per clone and per block were used.

### Phenotypic data

After harvest in autumn, a subset of the potato panel was evaluated for 18 phenotypic traits widely recorded in potato breeding programs in South America (CIP), Europe (Center for Genetic Resources the Netherlands) and North America (United States Potato Board). The traits averaged over both seasons were: percent of dry matter (DM, assessed by gravimeter method according to [23]), chip quality (CQ, visually scored according to [10]), tuber yield (TY, Tn/ha and gm/tuber), number of commercial tubers (CT), tuber aspect (AS, measured with a visual scale of 1 to 9), *Fusarium solani* infection (FR), *Streptomyces scabies* infection (SR), internal necrosis (IN, presence/absence), hollow heart (HH, presence/absence), occurrence of tuber cracks (TC, presence/absence), insect damage level (ID), skin type (ST), skin and flesh color (SC and FC respectively), tuber shape (S), eye depth (ED), tuber size (TZ) and a priori group (AG). The descriptors and scores used mainly correspond to the guidelines in international potato databases and current authors (www.europotato.org; http://www.cipotato.org/catalogue; http://potatoassociation.org/; [20]). FR, SR and ID were measured as slightly affected (less than 10% of tuber weight in less than 10% of the tubers); intermediate (more than 11% and less than 20% by weight of the tuber and between 11% and 20% of the tubers); and severely affected (more than 20% in weight in more than 20% of the tubers). TZ was measured as small (less than 50 mm); medium (between 51 mm and 150 mm) and large (more than 150 mm); and ED as shallow (less than 1 mm depth), intermediate (more than 1 mm and less 2 mm) and deep (more than 2 mm). ST, SC, FC and S were evaluated according to CIP (http://www.cipotato.org/catalogue). AS is determined by the visual observation of the local breeder according to the commercial preferences in the Argentine market; it integrates eye depth, skin type and size. Bacterial and fungal diseases in the experimental field during both growing seasons reduced the number of genotypes that were assessed phenotypically to a subset of 144 out of an initial set of 183 genotypes. The summary of scores for each phenotypic trait on 144 potato genotypes is presented in Additional file 2.

### DNA extraction

DNA was obtained from 0.1 g healthy and young leaf tissue. Total genomic DNA was extracted using DNeasy plant mini kit (QIAGEN, Hilden, Germany) according to supplier instructions. The concentration of the purified genomic DNA was adjusted to 50 ng*μL^−1^ using PicoGreen quantitation (Eugene, Oregon).

### SNP genotyping

The total of the potato panel (183 genotypes) was genotyped using the new Illumina Infinium 12 K V2 Potato Array [24, 25]. The V2 array contains 12,808 SNPs, the set of markers from the previous Infinium 8303 Potato Array and additional SNPs selected for genome coverage and on candidate genes and regions with resistant genes. The arrays were read using the Illumina iScan Reader with Infinium® HD Assay Ultra (Illumina Inc., San Diego, CA). Genome Studio 2011.1 software (Illumina, San Diego, CA) was used to assess initial sample quality. Tetraploid (5-cluster) genotyping was based on theta value thresholds, using a custom script from the Solanaceae Coordinated Agricultural Project-SolCAP [14]. Following 5-cluster calling, a filtering process was carried out to identify 4859 high-quality markers. These markers were used for further molecular analysis.

### Multivariate analysis of phenotypic traits

The association among phenotypic traits was measured using Pearson correlation coefficient to determine the variables to be used in the multivariate analysis. Subsequently, grouping of the 144 genotypes was performed by using three approaches: a) Principal Component analysis (PCA), conducted on standardized and significantly correlated quantitative variables (ED, AS, TZ, DM, CT, TY and CQ); b) Principal Coordinates analysis (PcoA) for the DICE coefficient of similarity between genotypes, graphed in a two-dimensional scatter plot, the DICE coefficient was calculated as d_ij_ = (1-S_ij_)^1/2^ where d_ij_ is the distance between element i and j, and S_ij_ is similarity coefficient of DICE [26] using significantly correlated qualitative and binary variables (AG, IN, HH, ID, SC, and FC); and c) a General Procrustes analysis (GPA) based on both types of correlated phenotypic traits [27]. A Minimum Spanning Tree (MST) from the corresponding distance matrix was added to PCA and PcoA. The genotypes were symbolised by different coloured dots both in PCA and PcoA plots, according to assigned a priori groups*.* The Euclidean distance between genotypes in the consensus space was calculated to illustrate the consensus configuration generated by GPA using an unweighted pair-group method based on average arithmetic algorithm (UPGMA). The cophenetic correlation was calculated to examine the adjustment of clustering [28]. The correlation analysis and plot was done in Genstat 18th edition (VSN International Ltd., Oxford, UK). PCA, PcoA and GPA were performed using Info-Gen software [29].

### Population heterozygosity and molecular diversity

The Illumina Infinium bi-allelic genotyping platform allows scoring up to five allele dosage genotype calls per SNP (AAAA, AAAB, AABB, ABBB, BBBB). These correspond to the expected genotype configuration for an auto-tetraploid species, compared to the three genotype calls found for a diploid species (AA, AB, BB). The population was divided into a priori groups according to previously features described (Additional file 1). The observed heterozygosity (Ho) was calculated as Ho = (AAAB + AABB + ABBB)/(AAAA + AAAB + AABB + ABBB + BBBB). Specific estimators for the mean number of alleles per locus (A/L), the mean number of effective alleles (AE) and unbiased Nei heterozygosity (He) [30] were assessed by a bootstrap procedure with 1000 permutation, using InfoGen/P software [29].

### Population phylogeny and structure

The phylogeny and structure of the potato population were calculated using the SNPs molecular marker information. For all genotypes, the phylogeny was determined by MST using the Prim’s algorithm [31]. Different coloured dots symbolised the genotypes according to assigned a priori groups.

Population sub-structures among the 183 genotypes were determined using the STRUCTURE 2.3 software [32], adopting the admixture model with correlated allele frequency. The program was run for hypothetical subgroups ranging from one to ten (K values). Runs were carried out by pre-setting 10^6^ iterations, of which only the first 10^5^ were not recorded (burn-in period). Each run was replicated three times, and the population structure was a result of a consensus. Then, Evanno transformation method was used to infer the most likely number of populations (K) [33]. STRUCTURE PLOT program [34] was utilised to display the graphics of population structure.

Additionally, a PCA analysis was performed on genotypes score of SNPs data as described by Uitdewilligen et al. [35]

The consensus configuration generated by the UPGMA dendrogram was contrasted to the grouping by genetic background (STRUCTURE plot) and to the a priori grouping for the same genotypes to check for concordance between genotypic and phenotypic data.

## Results

### Grouping genotypes according to quantitative traits

**Fig. 1:**
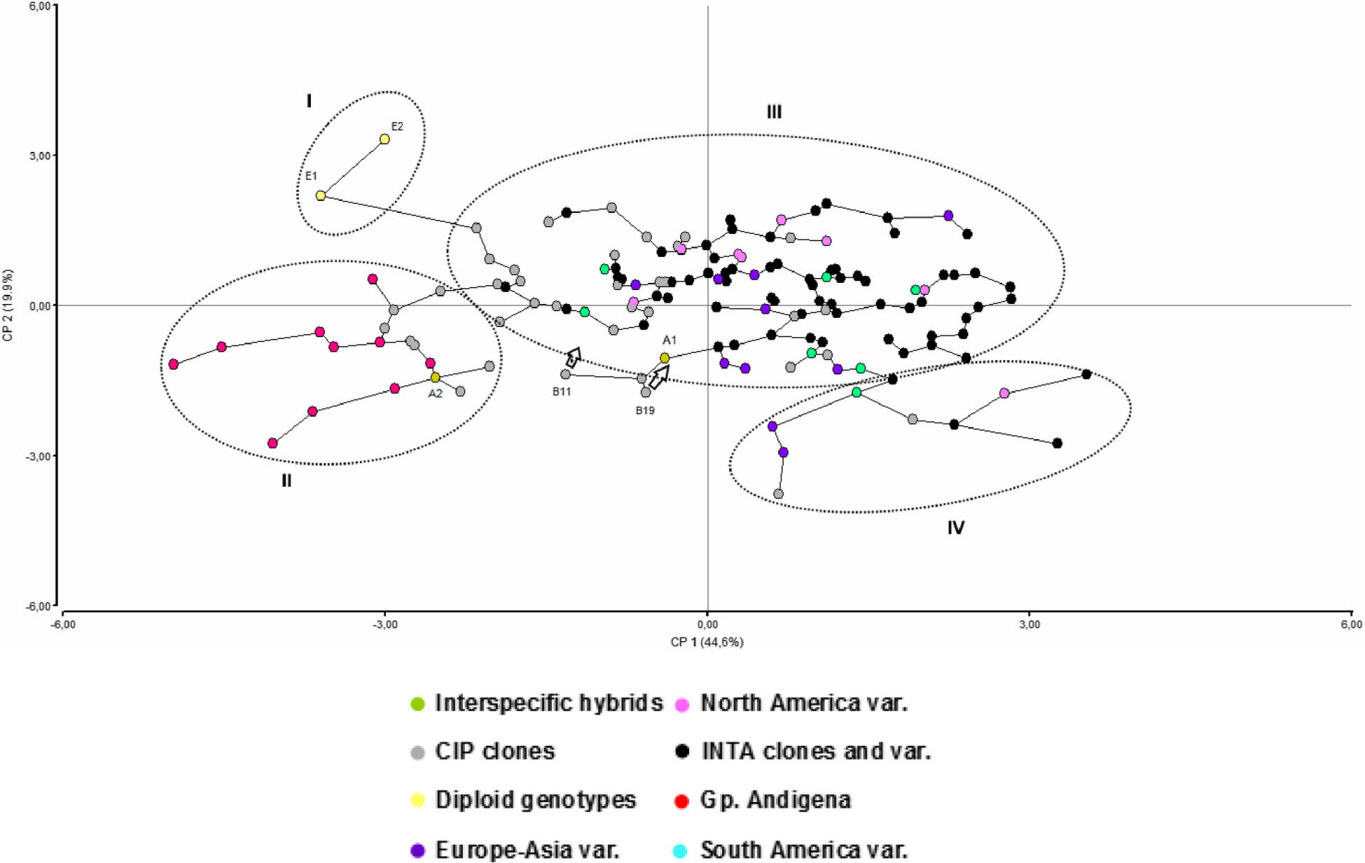
Principal Component Analysis (PCA) for quantitative traits on 144 potato individuals. Axis 1 (48, 4%) and Axis 2 (16, 9%) explained 65% of total variation. Minimum Spanning Tree corresponding to the distance matrix was added. Each dot symbolises a unique genotype and the initial letter in the name the a priori group affiliation. Dot colors corresponded to a priori group classification as follows: green (interspecific hybrids), grey (CIP clones), pink (North America varieties), black (INTA clones and varieties), yellow (diploid genotypes), red (Group Andígena), violet (Europe-Asia varieties), turquoise (South America varieties)

The PCA analysis of standardised log transformation and significantly correlated (at 0.001 level) quantitative phenotypic traits (TY, CT, AS, DM, CQ, ED and TZ) for the 144 potato genotypes is shown in Fig. 1. The first three main eigenvalues on the Euclidean similarity matrix explained 44.6, 19.9 and 11.89% of the total variation, respectively. According to the location in the graph, the genotypes tended to gather in four groups. The ordination was mainly determined by yield related traits on axis one (eigenvalue of TY: 0.51, eigenvalue of CT: 0.5 and eigenvalue of TZ: −0.48, increasing to the right) and, to a lesser extent by industrial quality related traits on axis two (DM and CQ aptitude increasing downward). The larger group (III-group) included genotypes of a priori groups designated as INTA-Balcarce clones (black dots), a smaller number of CIP clones (grey dots) and Europe-Asian, South and North American commercial clones (violet, turquoise and pink dots, respectively). Smaller groups involved *S. tuberosum* Gp. Andígena clones (red dots) and CIP clones (II-group), diploid clones (yellow dots) (I-group) and a complex set of European-Asian commercial clones, INTA-Balcarce clones and CIP clones (IV-group). The two hybrid genotypes derived from a cross between *S. tuberosum* Gp. Tuberosum var. Calen INTA and *S. gourlayii* (A-named genotypes) were located in different groups (A1 in III-group and A2 in II-group). The B19 and B11 CIP clones were considered as part of the III-group because of having the smallest distance with members of that group in the corresponding MST.

### Grouping genotypes according to qualitative traits

**Fig. 2:**
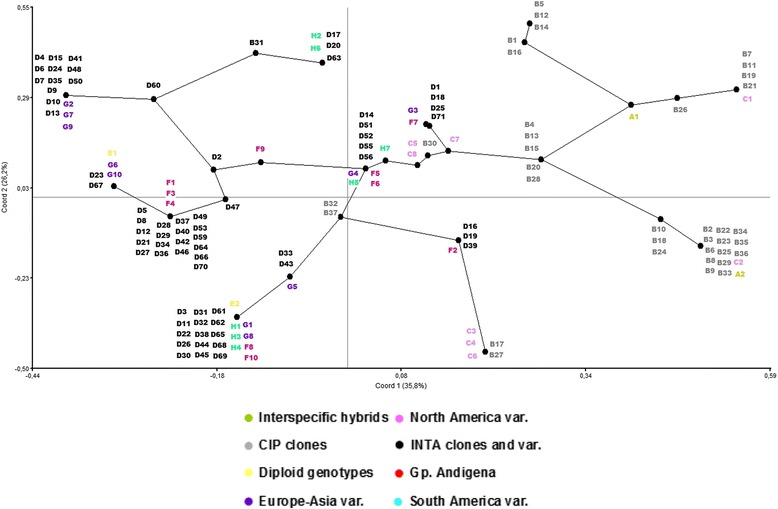
Principal Coordinate analysis (PcoA) for qualitative traits. The spatial classification of 144 genotypes of potato was depicted in two first coordinates. Minimum Spanning Tree was added according to distance matrix. Colored capital letters indicate a priori groups as follows: green (interspecific hybrids), grey (CIP clones), pink (North America varieties), black (INTA clones and varieties), yellow (diploid genotypes), red (Group Andígena), violet (Europe-Asia varieties), turquoise (South America varieties). Numbers indicate genotypes. Each dot indicates several co-located genotypes

Figure 2 shows the PcoA analysis of the 144 genotypes for six significantly correlated (at 0.001 level) qualitative phenotypic traits (AG, IN, HH, ID, SC, and FC). The first three coordinates, explained the 35.8, 26.2 and 18.1% of the total variation, respectively. Genotypes tended to order mainly across the first coordinate generating two groups, predominantly related to a priori group and skin colour as the more discriminant variables for this analysis. The group on the left side was represented mainly by INTA-Balcarce clones (black labels) and the group on the right side by CIP clones (grey labels). Minor a priori groups (F, C, and H) were located between the both major ordination groups, except varieties of European-Asian origin (violet labels) and diploid genotypes (yellow labels) that were located together with INTA-Balcarce clones. On the other hand, interspecific genotypes (A-named genotypes, green labels) were assigned to the CIP group of clones.

### Assessment of genetic diversity with SNPs markers

Observed and expected heterozygosity (Ho and He, respectively) of the 183 genotypes population is shown in Table 1. The mean observed heterozygosity of the population was 0.51 ± 0.062, and the mean expected heterozygosity was 0.52 ± 0.01. In general, the observed heterozygosity was greater than the expected heterozygosity in the different a priori groups. The a priori groups within *S. tuberosum* Gp. Tuberosum showed values of observed heterozygosity of 0.6 approximately and in all cases higher to the expected heterozygosity. However, the observed heterozygosity values for the 11 genotypes of *S. tuberosum* Gp. Andígena (F) presented a lower estimation (Ho = 0.43 ± 0.033) in comparison with *S. tuberosum* Gp. Tuberosum (Ho = 0.61 ± 0.02) (a priori groups B, C, D and G; Table 1). The two interspecific hybrid genotypes presented average values of observed heterozygosity between that of *S. tuberosum* Gps. Andígena and Tuberosum (0.537 ± 0.07) and a lower expected heterozygosity (0.327 ± 0.02). The two diploid genotypes (E) resulted in the minimal and very similar heterozygosity values (He = 0.046 ± 0.01 and Ho = 0.04 ± 0.01).

**Table 1:** Summary of genetic diversity of potato accessions from diverse provenance sources, species and ploidy levels

A Priori group	Source	Species	N gen	A/L	AE	He	Ho
A	INTA^1^, NWA^2^	Interspecific hybrid^3^	2	1.56 ± 0.03	1.34 ± 0.02	0.327 ± 0.02	0.537 ± 0.07
B	CIP^4^	*S. tuberosum* Gp. Tuberosum	49	2 ± 0.01	1.71 ± 0.02	0.47 ± 0.01	0.592 ± 0.056
C	NA^7^	*S. tuberosum* Gp. Tuberosum	9	1.99 ± 0.01	1.71 ± .0.02	0.48 ± 0.01	0.654 ± 0.041
D	INTA^1^	*S. tuberosum* Gp. Tuberosum	83	2 ± 0.02	1.77 ± 0.01	0.49 ± 0.01	0.618 ± 0.04
E	NWA^2^	*S. chacoense* *S. tarijense*	2	1.04 ± 0.01	1.04 ± 0.01	0.04 ± 0.01	0.046 ± 0.001
F	SA^5^	*S. tuberosum* Gp. Andígena	11	1.68 ± 0.03	1.37 ± 0.02	0.42 ± 0.02	0.429 ± 0.033
G	EA^6^	*S. tuberosum* Gp. Tuberosum	19	1.98 ± 0.01	1.61 ± 0.02	0.43 ± 0.01	0.604 ± 0.031
H	SA^5^	*S. tuberosum* Gp. Tuberosum	8	1.94 ± 0.01	1.57 ± 0.02	0.42 ± 0.01	0.601 ± 0.017
Total genotypes			183	2 ± 0.01	1.85 ± 0.01	0.517 ± 0.01	0.51 ± 0.062

^1^INTA Balcarce

^2^North West provinces of Argentine

^3^*S. tuberosum* Gp. Tuberosum var. Calen INTA x *S. gourlayii*

^4^International Potato Center

^5^South American countries

^6^Europe-Asia countries

^7^North American countries; N° gen.: number of genotypes analyzed; A/L: mean number of allele per locus; AE: mean number of effective allele; He: unbiased mean expected heterozygosity of Nei; Ho: observed heterozygosity. Potato genotypes were grouped in a priori *groups* according to the taxonomy, ploidy level, breeding program and geographic provenance

### Population phylogeny obtained from molecular data

**Fig. 3:**
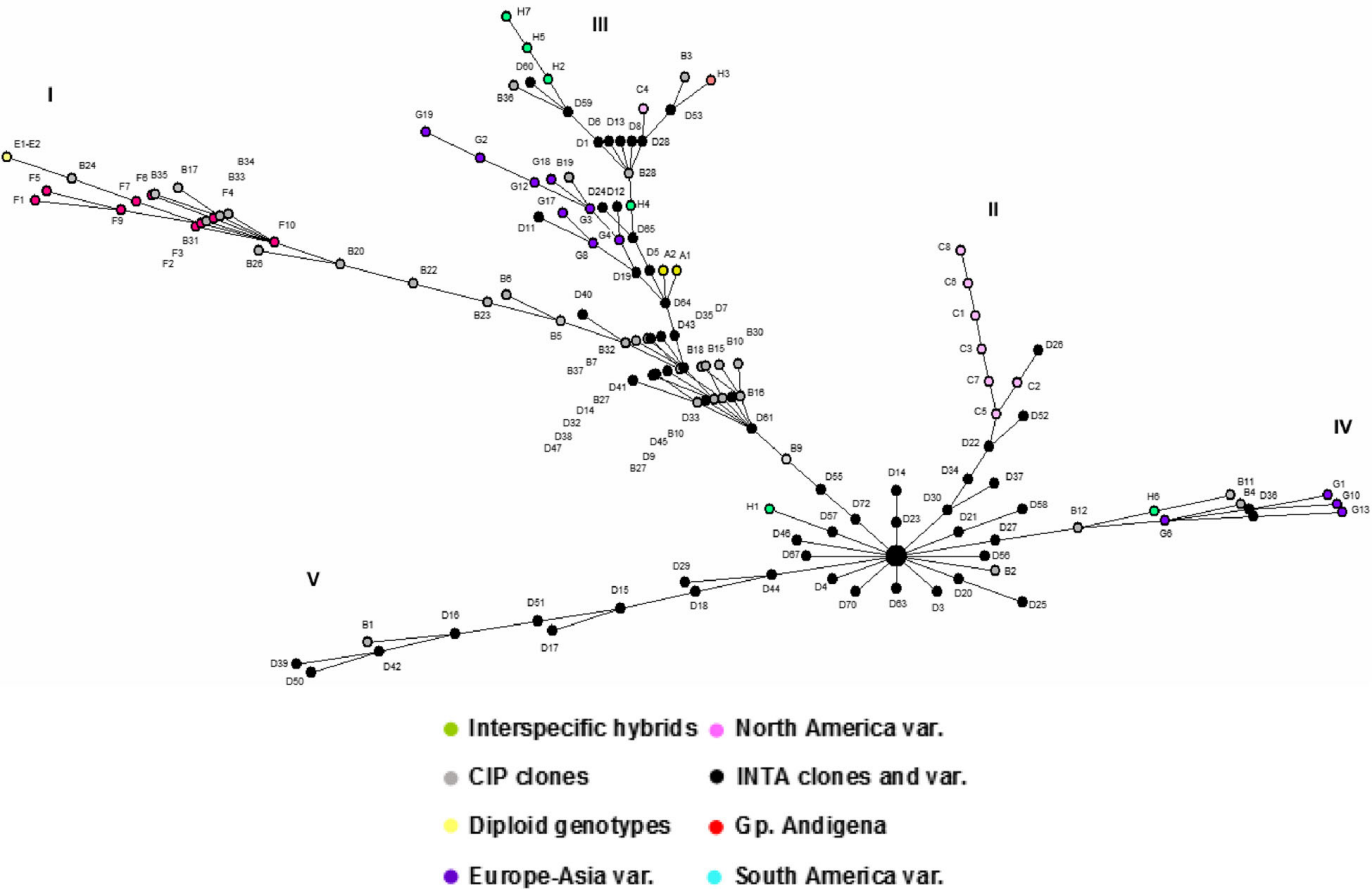
Inferred population Phylogeny. Minimum spanning tree (MST) based on 4859 high-quality SNPs using the Prim′s algorithm. Huinkul-MAG was set as root genotype. Each dot symbolizes a different genotype and its color a priori group as follows: green (interspecific hybrids), grey (CIP clones), pink (North America varieties), black (INTA clones and varieties), yellow (diploid genotypes), red (Group Andígena), violet (Europe-Asia varieties), turquoise (South America varieties)

The MST obtained using the Prim’s algorithm (Fig. 3) suggested the presence of a structured population. Huinkul-MAG was set as root genotype because it is a parent related with the majority of INTA-Balcarce a priori group (the largest group). The Andígena group varieties and diploid wild genotypes stood out in a separate group (I). In the same way, North American clones took a single branch (II). As expected, INTA-Balcarce clones and INTA varieties were closer to Huinkul-MAG genotype. The CIP and INTA-Balcarce clones mostly shared branches, showing the linkage between both potato breeding programs. South American and European-Asian varieties shared branches (III and IV) showing the links between them, unlike to what was observed with the Andígena and North American groups. It is important to note that CIP and INTA-Balcarce clones were spread among all branches in different degrees, mainly in a non-terminal position. INTA-Balcarce clones mostly integrate the (V) branch, showing that in the several years of this potato breeding program, some elite clones were used as parents to new offspring in a recurrent selection scheme.

### Population structure determined by Bayesian approach

**Fig. 4:**
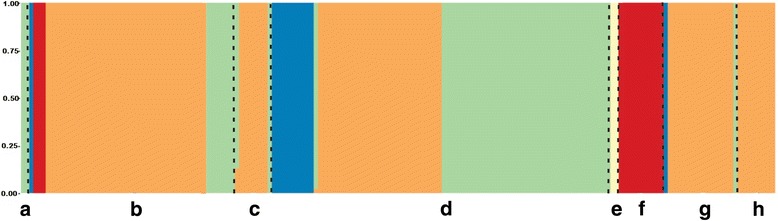
Inferred population structure. Bar plot based on 4859 Single Nucleotide Polymorphism (SNP) markers generated by STRUCTURE 2.3 using the admixture model. Each colour represents a cluster (K = 5). Each column (183 columns in total) represents a potato genotype, which is partitioned into segments. Each segment represents the estimated contribution of the participating genetic background (cluster). The a priori groups are indicated capital letters (A-H) as described in Table 1

From Evanno et al. [33] methodology, delta K vs. K plot showed five groups as an adequate solution (K = 5) (Additional file 3). In Fig. 4, the sub population structure bar plot for the 183 columns (a column represented each genotype) showed that more than 95% of the columns were strongly assigned to a single ancestral genetic background, each displayed by a different colour. When the a priori groups were compared with the STRUCTURE grouping (K) (Fig. 4), the Andígena group (F) was observed to be linked to a single genetic background (red colour). The CIP (B) and INTA-Balcarce (D) clones shared three genotypic backgrounds (blue, orange and green colours), the last two were the most numerous. These observations were coincident with those represented in Fig. 3, in which both a priori groups presented genetic closeness. The North American, European-Asian and South American varieties (C, G, H a priori groups, respectively) expressed highly similar genetic background (primarily genetic background coloured in orange). As expected, diploid individuals possessed a unique genetic background (pale yellow colour). Interspecific hybrids genotypes (A) presented a genetic background (green colour) also found in other a priori groups, primarily CIP (B) and INTA-Balcarce (D) a priori groups.

### Joint analysis: Grouping by molecular and phenotypic traits

**Fig. 5:**
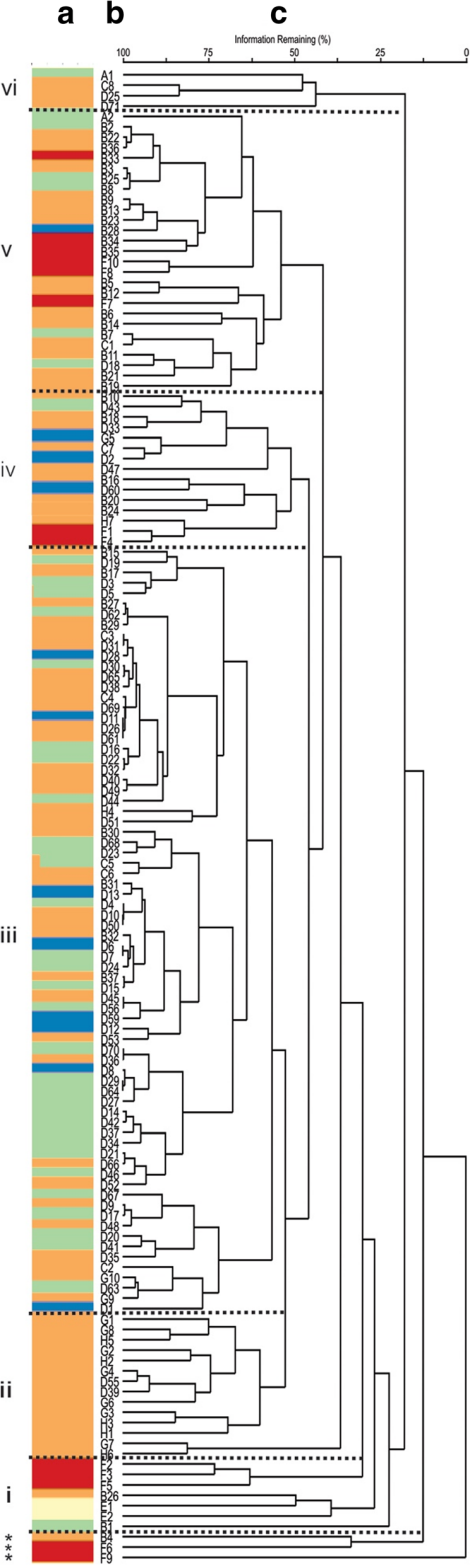
Joint analysis between Generalized Procrustes Analysis (GPA) and STRUCTURE of 144 potatoes genotypes. a. Bar plot of population structure by STRUCTURE; each genotype is represented by a horizontal bar: Each segment of the horizontal bar represents the estimated contribution of the participating genetic backgrounds (clusters) (i-vi). b. Labels who represent a priori groups and genotypes. c. Dendrogram using Euclidean distance for consensus configuration obtained by General Procrustes Analysis (GPA) with quanti-qualitative descriptors and unweighted pair-group method using arithmetic averages (UPGMA) grouping method

Figure 5 shows the STRUCTURE plot combined with the UPGMA dendrogram derived from GPA. The STRUCTURE plot is broken in five segments colour code differentiated along Y-axes (Fig. 5 a). Each genotype was represented by a single horizontal bar in the plot. Based on a standardised consensus of phenotypic traits, the dendrogram showed six groups (i-vi) with a cophenetic distance of 0.85 (Fig. 5c). Outlier genotypes were in the cluster indicated with asterisks. By comparing these groups with the a priori grouping (Fig. 5b), a linkage among phenotypic classification, a priori groups and genetic background was observed in most cases. Indeed, diploid and *S. tuberosum* Gp. Andígena genotypes were located similarly in the i-cluster by phenotypic and genetic data, (pale yellow and red colours, respectively). European-Asian and South-American varieties were located mainly in the ii-cluster and were related only to worldwide commercial potato varieties (orange colour). The breeding program clones were placed mostly in a different cluster. The most numerous cluster (iii-cluster) was formed by INTA-Balcarce clones followed by a few CIP and worldwide commercial potato genotypes, they share genetic background associated with worldwide commercial varieties and interspecific hybrid genotypes (orange and green colours, respectively). On the other hand, most of CIP clones were mainly located in the v-cluster with a few *S. tuberosum* Gp. Andígena genotypes that were somewhat related to worldwide commercial varieties and interspecific hybrids (red, orange and green colours, respectively). A minor dendrogram cluster made from phenotypic characters (iv-cluster) contained clones from both breeding programs with a few genotypes belonging to *S. tuberosum* Gp. Andígena and commercial varieties. Once again, the genetic background associated with worldwide commercial varieties (orange and blue colours) and *S. tuberosum* Gp. Andígena genotypes (red colour) shared a phenotypic cluster. Finally, vi-cluster gathered only four different genotypes with the majority of worldwide commercial varieties (orange colour).

## Discussion

Currently, the global potato demand for improved varieties is increasing, forcing breeders to focus on the development of varieties resistant to biotic and abiotic stresses but maintaining high yields, good tuber aspect and excellent quality. Therefore, having a genetically diverse germplasm in the early stages of potato breeding is essential. A priori clustering was generated for a panel of diverse germplasm (diploid, interspecific hybrids, South-America, North American, European-Asian varieties, CIP clones and local breeding program clones), and showed to be determinant in assessing the diversity and structure of the population.

### Phenotypic diversity

The spatial classification of a priori grouping of genotypes using phenotypic traits showed that grouping depended on the characters considered to the analysis. PCA with quantitative traits could readily distinguish diploid genotypes on the one hand and those belonging to *S. tuberosum* Gp. Andígena potatoes (Fig. 1). Diploid (wild) genotypes are described as defective in traits related to yield and industrial interest because they are mainly included in breeding programs as a source of biotic/abiotic stress tolerance genes [12, 36]. Therefore, they were considered as outlier genotypes and as a measurement of the quality of the cluster methodology. At the same time, previous authors described the *S. tuberosum* Gp. Andígena varieties as a genetic pool for short day adapted genotypes, subject to local farmers breeding [37]. The PCA was not able to separate the most worldwide commercial varieties and breeding program genotypes, independently of the source. The long-day adapted genotypes were mainly situated on the graph regions linked with high yield and moderate industrial aptitude. D’hoop et al. [18] reported that strong selection pressure for yield-related and moderated selection pressure for quality traits (dry matter and chipping colour) have played a leading role in the design of the current commercial potato germplasm. In fact, elite European-Asian varieties are the result of decades of breeding for market traits as the ones used in this work [38, 39], and consequently they are of great interest as parents in the breeding programs. However, *S. tuberosum* Gp. Andígena and South American varieties are local elite potatoes and hence are adapted to their local environment. INTA-Balcarce selected genotypes for industrial use, yield and tuber aspect characters (high yield, light skin and flesh colours, oval shape, etc.) under long days, meanwhile CIP breeding programs included biotic and abiotic stress expanding the range of colours and shape potatoes adapted to short days.

When easy measurable qualitative traits mediated the grouping, the source of the potato breeding program was the major cause for grouping (Fig. 2). INTA and CIP breeding programs are recognised for having a highly diverse potato germplasm [37]. Nevertheless, the CIP germplasm is constituted by a higher proportion of *S. tuberosum* Gp. Andígena germplasm than the INTA-Balcarce breeding program. Hence, traits like deep eyes, coloured skins and coloured flesh distinguished and separated the materials originated from CIP. The INTA-Balcarce breeding program has prioritised cooking quality traits in the selection process as required by the growing local processing industry; similar breeding process occurs in European breeding programs [23].

### Molecular diversity

The germplasm heterozygosity estimated for all genotypes included in a priori groups showed moderate high values, both in observed and Nei heterozygosity (in average greater or equal to 0.5) (Table 1). These results are coincident with previous authors that describe the INTA-Balcarce breeding and the germplasm bank as a high genetic variability resource [36, 40]. The a priori groups showed different levels of heterozygosity (Table 1). While several authors describe a narrow genetic diversity in cultivated potato varieties [41, 42], the levels of heterozygosity of *S. tuberosum* Gp. Tuberosum were much higher than the rest of the tetraploid and diploid individuals, in agreement with Hirsch et al. [14]. The highly diverse origin of the clones of Gp. Tuberosum studied here, with few common ancestors is the main reason for this high heterozygosity [43]. The low values of heterozygosity in *S. tuberosum* Gp. Andígena genotypes were also unexpected because it has been described as a group with high genetic diversity [44]. Recently, similar observations of low heterozygosity were found by Hirsch et al. [14]. The samples studied here correspond to a small subsample of the existing Gp. Andígena genetic diversity, and it is restricted to ten genotypes of Argentine origin. Moreover, the Infinium SNPs array was created including SNPs with no other polymorphisms within 100-bp windows and detected on long day adapted potatoes. On the one hand, the larger amount of allelic combinations and allelic dosage in the tetraploid ploidy level allows achieving more variability in comparison to diploid species.

### Phylogeny and population structure

Both breeding potato programs (CIP and INTA-Balcarce) were located at separate branches (Fig. 3). CIP germplasm was associated with a higher proportion of *S. tuberosum* Gp. Andígena varieties and diploid genotypes, while INTA-Balcarce germplasm showed an inverse proportion of these individuals. This observation confirms the diverse genetic origin of INTA potato germplasm.

The closeness between South-America and European- Asian varieties in Fig. 3 is related to the fact that older European potato genotypes were selected as parents of younger South American varieties ([45]; www.europotato.org; www.inia.cl; www.inta.gob.ar/variedades).

North American cultivated potatoes are associated with a narrow genetic diversity due to the utilization of cultivar Katahdin as a progenitor. Almost a quarter of the germplasm that originated the elite cultivars in North America has Katahdin on its the genetic background [41, 46]. Therefore, the separate North American branch reveals a closer kinship among the North American potato varieties.

When genetic clustering analysis by SNPs markers was assessed through Bayesian approach [11], the evaluated germplasm showed a strong population structure, with five different genetic backgrounds (Fig. 4). It is remarkable that the potato varieties included within a priori group (A to H) had fairly homogeneous genetic background. Each a priori group represents potato genotypes from a common breeding source, ploidy level, and taxonomic classification, hence shared similar phenotypic trait selection in breeding programs. A priori groups associated with North America, South America and European-Asian varieties (C, H and G, respectively) revealed practically the same genetic background according to STRUCTURE analysis (orange colour) (Fig. 4). It was consistent with the results of the phylogeny analysis by Prim’s algorithm (Fig. 3). These results agree with the already described history of human driven gene migration of potato [45]. In the same line, the genetic background associated with blue colour could represent European-Asian varieties whose genetic background was included as parental lines in the CIP and INTA-Balcarce germplasm. The interspecific genetic background (*S. tuberosum* Gp. Tuberosum var. Calén x *S. gourlayii*) (green coloured) found mainly within INTA-Balcarce genotypes pointed out the clones having hybrid genomes. A non Hardy-Weinberg equilibrium based on Discriminant Analysis of Principal Components (DAPC) was performed with the same SNPs data (Deperi et al., unpublished data). Similar population structure was observed in both approaches. A PCA using SNPs was also done, and it was observed similar groupings to the PCA with the phenotypic traits (Additional file 4).

Differences between phylogeny tree and STRUCTURE analysis may be due to the requirement of setting a root genotype in the former, which could render a bias that is not present in the Bayesian cluster. The Bayesian clustering method was able to demonstrate the ancestral genetic structuring inherent to the germplasm under study. While different genetic backgrounds are represented in different proportions (reflected by each colour), both germplasm pools had a high allelic diversity, as previously described (http://cipotato.org/es/; http://inta.gob.ar/balcarce).

CIP, INTA germplasm and North and South American varieties showed a significant presence of European-Asian alleles. It is well documented that the long day adapted elite European varieties were broadly spread in America by local farmers in part due to their high yields and attractive external appearance [45, 46]. Elite European varieties have been gene donors for several decades in several breeding programs [14, 36]. On the other hand, the diploid species were outlier genotypes as expected from their genetic background: lack of genetic interactions originated from natural selection and with many undesirable commercial characteristics.

### Joint characterization by multivariate and molecular analysis

Previous authors demonstrated that major phenotypic differences could often occur with minor genotypic changes [47]. Molecular markers, such as SNPs, assess minimal changes in crop genomes enabling the association between phenotypic and genotypic traits in potato [12, 16, 18].

Simultaneous clustering characterization between both types of phenotypic traits carried out by GPA and then aligned to STRUCTURE figure (Fig. 5) added significant value to this analysis. Bramardi et al. [1] described that the dissimilar nature of variables must be considered to assess an accurate consensus configuration. Therefore, GPA arises as a tool able to characterise genotypes measured by quantitative and qualitative traits. Consensus configuration improved PCA and PcoA analyses. Consensus clustering was able to distinguish genotypes and groups that in the a priori classification were considered dissimilar (e.g.: *S. tuberosum* Gp. Andígena to commercial varieties, or genotypes of each breeding program) (Fig 5c).

*S. tuberosum* Gp. Andígena genotypes that were located alone in PCA were also in a separate branch in the dendrogram (e.g.: Morada Morada (F6) and Tuni Blanca (F9) genotypes). The *S. tuberosum* Gp. Andígena clones were selected under short day high mountain habitats in contrast to *S. tuberosum* Gp. Tuberosum clones, showing a distinct genetic background with suboptimal response for yield and industrial traits at INTA Balcarce long day assays [37]. This repeated behaviour strengthens the value of local breeding efforts done by South American farmers [36, 37]. When comparing with PCA and PcoA analysis separately, it could be observed that only GPA configuration could gather European-Asian and South American varieties bearing a homogeneous genetic background.

On the other hand, as shown with PcoA (Fig. 2), the GPA phenotypic dendrogram showed the relationships between CIP and INTA breeding programs. Most of INTA-Balcarce/North American and CIP/*S. tuberosum* Gp. Andígena genotypes were mainly placed into two separate clusters in the dendrogram (Fig. 5). When relating these clusters with the genetic background, the genetic links among them are evident. Therefore, only a certain part of the genetic background was shared between these two breeding programs and the differential phenotypic selection in each breeding program defines the major differences among these clones.

## Conclusions

The phenotypic evaluation with easily measured traits could be an effective and a low-cost strategy in preliminary grouped breeding populations if variables with acceptable discriminant capacity are used. The most effective discriminant variables used in this study were yield, the number of commercial tubers, tuber size, dry matter, frying ability, a priori group, and skin colour.

PCA based on quantitative traits enabled discrimination among diploid, Gp. Andígena and Gp. Tuberosum. Additionally, the PcoA approach was only able to discriminate between the local and CIP breeding programs. However, a GPA phenotypic dendrogram with the consensus PCA and PcoA clustering was able to make a better discrimination. As supported by the SNP analysis presented herein, a priori grouping of genotypes based on the source, ploidy level and the taxonomic category, was an accurate strategy for preliminary ancestral clustering of genetically similar breeding material.

SNPs confirmed the presence of clusters within the potato population. The phylogeny and cluster analysis performed using STRUCTURE demonstrated the significant introgression of Elite-European potato germplasm into several a priori groups of this panel. The potato genetic diversity identified in this work showed the potential of incorporating these genotypes in further association mapping studies.

In cases where little information on the parental material is available, a multi-character screening with yield, the number of commercial tubers, tuber size, dry matter, frying ability, and skin colour will be useful for assembling a preliminary crossing panel with high heterozygosity. Later in the breeding process, a genetic analysis will be necessary to achieve a more accurate parental selection.

Considering previous observations related to the crossing history in potato varieties over the last centuries, the genotypes in this potato panel provide a good evidence of the linkages among the different sources of potato germplasm for breeding, and they showed the high degree of breeder-driven gene flow.

## Additional files

Additional file 1: Summary of potato genotypes evaluated by different approaches. For each genotypes evaluated, the name, a priori group which is linked, source of germplasm, ploidy level and type of screening performed are detailed. ^1^North West provinces of Argentine. (XLS 61 kb)

Additional file 2: Summary of scores for phenotypic traits on 144 potato genotypes. The scores for phenotypic traits: AG(a priori group), TY (tuber yield), CT (number of commercial tuber), AS (tuber aspect), FR (*Fusarium solani* infection), SR (*Streptomyces scabies* infection), IN (internal necrosis), HH (hollow heart),ID (insect damage level), TC (occurrence of tuber crack), ST (skin type), SC (skin color), FC (flesh color), S(tuber shape), ED (eye depth), TZ (tuber size), CQ (chip quality) and DM (dry matter) are presented for 144 potato genotypes order by its index label. (XLSX 24 kb)

Additional file 3: Delta K vs. K plot. According to the Evanno transformation method, the most likely number of populations (K) was inferred (Evanno et al. 2005). “DeltaK = mean(|L"(K)|) / sd(L(K))”. (PNG 24 kb)

Additional file 4: Principal Component Analysis from SNPs markers. Axis 1 (10%) and Axis 2 (5, 9%) explained 16% of total variation. Minimum Spanning Tree corresponding to the distance matrix was added. Each dot symbolized a unique genotype. Dot colors symbolize the a priori grouping as follows: green (interspecific hybrids), grey (CIP clones), pink (North America varieties), black (INTA clones and varieties), yellow (diploid genotypes), red (Group Andígena), violet (Europe-Asia varieties), turquoise (South America varieties). (PNG 16 kb)

## Abbreviations

A/L: Alleles per locus
AE: Effective alleles
AG: a priori group
AS: Tuber aspect
CIP: International Potato Center
CQ: Chip quality
CT: Number of commercial tuber
DAPC: Discriminant Analysis of Principal Components
DM: Dry matter
ED: Eye depth
FC: Flesh color
FR: *Fusarium solani* infection
GPA: General Procrustes Analysis
He: Expected heterozygosity
HH: Hollow heart
Ho: Observed heterozygosity
ID: Insect damage
IN: Internal necrosis
INTA: Argentine National Agricultural Technology
MST: Minimum spanning tree
PCA: Principal Component Analysis
PcoA: Principal Coordinates analysis
QTL: Quantitative Trait Loci
S: Tuber shape
SC: Skin color
SNP: Single Nucleotide Polymorphism
SR: *Streptomyces scabies* infection
ST: Skin type
TC: occurrence of tuber crack
TY: Tuber yield
TZ: Tuber size
UPGMA: Unweighted Pair-Group Method based on Average Arithmetic Algorithm
