# Supplementary figures and images for: Use of easy measurable phenotypic traits as a complementary approach to evaluate the population structure and diversity in a high heterozygous panel of tetraploid clones and cultivars
Source: BMC Genet. 2018 Jan 16;19:8. doi: 10.1186/s12863-017-0556-9 (PMC5771038; doi:10.1186/s12863-017-0556-9)

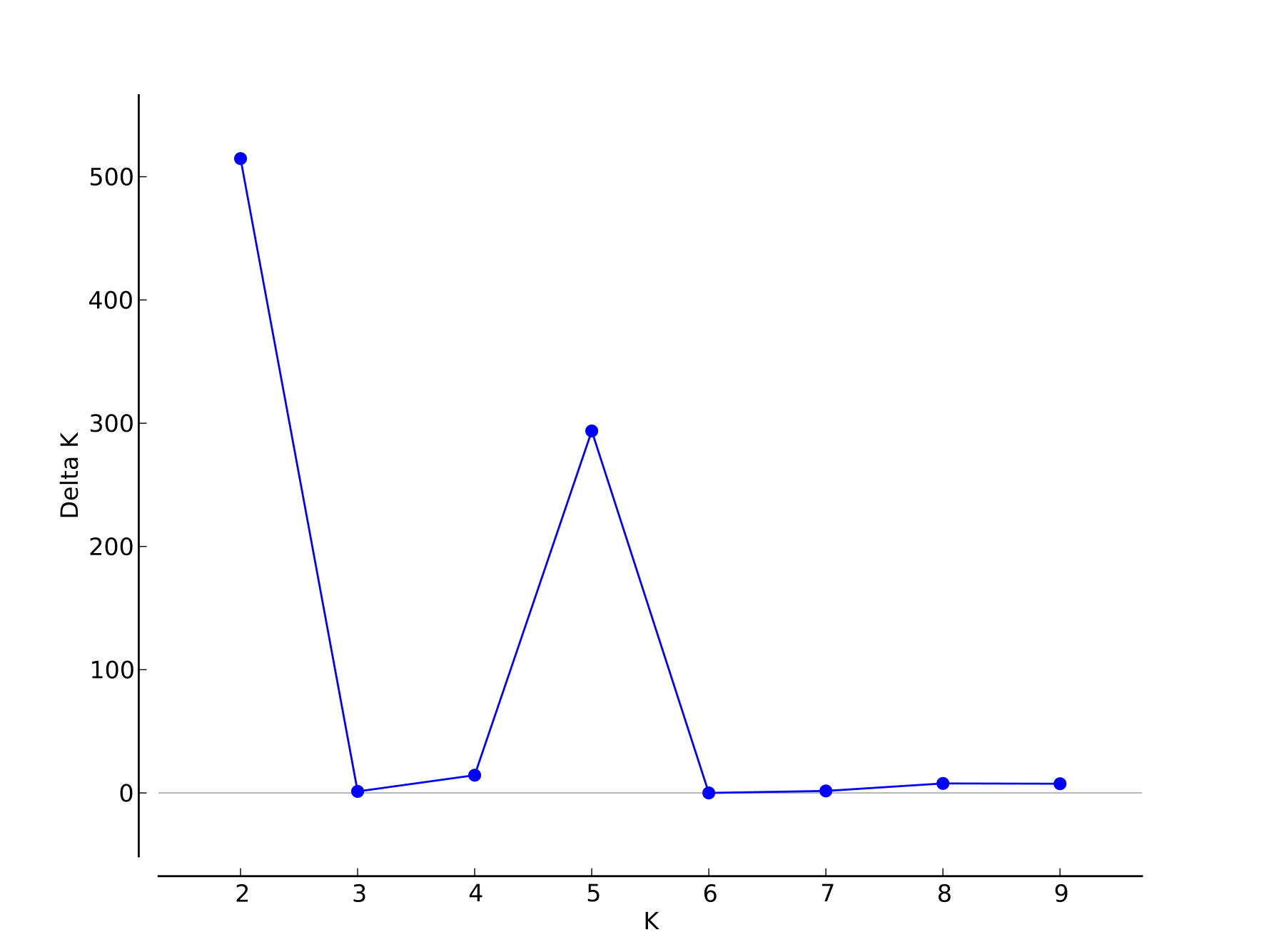

Supplement: Supplementary file 3 — Delta K vs. K plot. According to the Evanno transformation method, the most likely number of populations (K) was inferred (Evanno et al. 2005). “DeltaK = mean(|L"(K)|) / sd(L(K))”. (PNG 24 kb) [file 12863_2017_556_MOESM3_ESM.png]

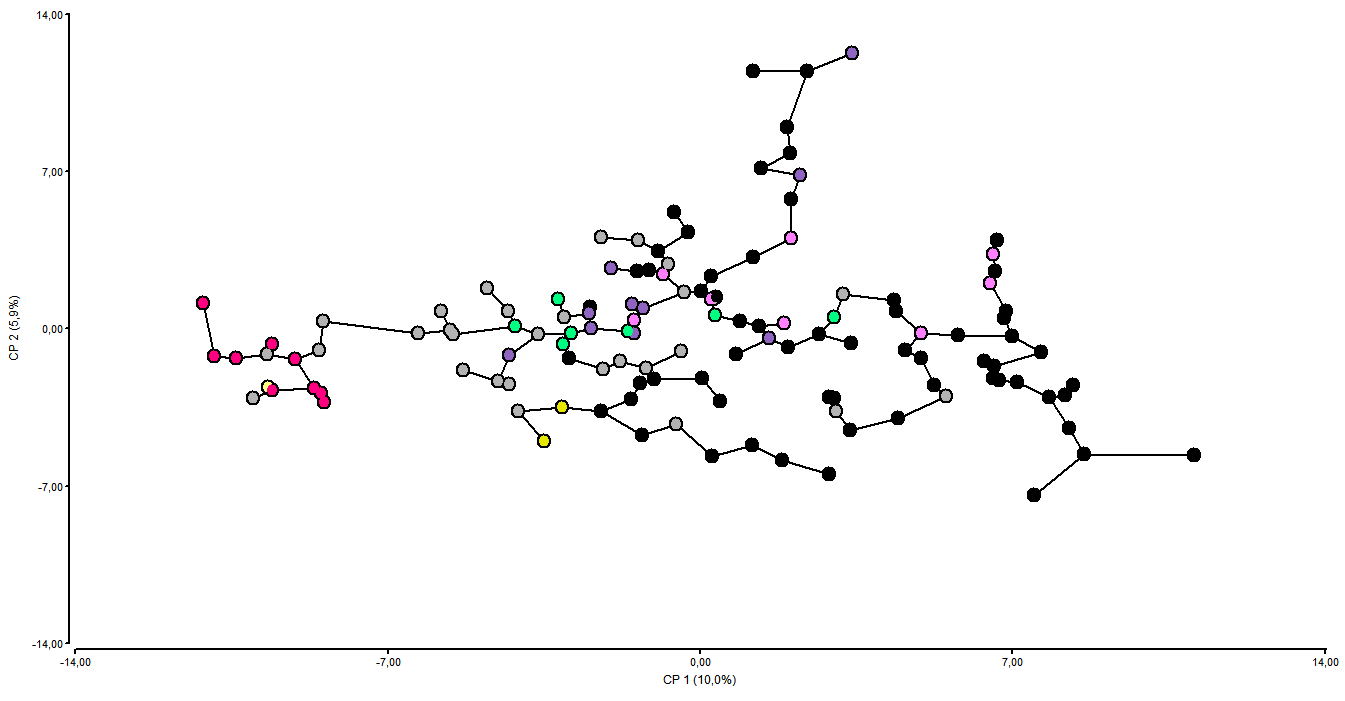

Supplement: Supplementary file 4 — Principal Component Analysis from SNPs markers. Axis 1 (10%) and Axis 2 (5, 9%) explained 16% of total variation. Minimum Spanning Tree corresponding to the distance matrix was added. Each dot symbolized a unique genotype. Dot colors symbolize the a priori grouping as follows: green (interspecific hybrids), grey (CIP clones), pink (North America varieties), black (INTA clones and varieties), yellow (diploid genotypes), red (Group Andígena), violet (Europe-Asia varieties), turquoise (South America varieties). (PNG 16 kb) [file 12863_2017_556_MOESM4_ESM.png]
